# Removal of Carbon Nanotubes from Aqueous Solutions by Sodium Hypochlorite: Effects of Treatment Conditions

**DOI:** 10.3390/toxics9090223

**Published:** 2021-09-16

**Authors:** Mei Yang, Toshiya Okazaki, Minfang Zhang

**Affiliations:** CNT-Application Research Center, National Institute of Advanced Industrial Science and Technology (AIST), Central 5, 1-1-1 Higashi, Tsukuba 305-8565, Japan; m-yang@aist.go.jp (M.Y.); toshi.okazaki@aist.go.jp (T.O.)

**Keywords:** carbon nanotubes, sodium hypochlorite, wastewater treatment, degradation, environmental safety

## Abstract

The treatment of carbon nanotubes (CNTs) containing wastewater has become an important issue with increasing industrial application due to the risk CNTs may pose to the environment and human health. However, an effective method for treating wastewater containing CNTs has not been established. Recently, we proposed a method to remove CNTs from aqueous dispersions using sodium hypochlorite (NaClO). To explore the practical applications of this method, we herein investigate the influence of different conditions, such as NaClO concentration, reaction temperature, pH value, and CNT concentration, on the CNT degradation rate. The results showed that the degradation of CNTs depends strongly on temperature and NaClO concentration: the higher the temperature and NaClO concentration, the faster the degradation rate. The optimal temperature and NaClO concentration are 50–70 °C and 2–3 wt%, respectively. Lower pH accelerated the degradation rate but induced the decomposition of NaClO. Furthermore, dispersants and other substances in the solution may also consume NaClO, thus affecting the degradation of CNTs. These findings are of significance for establishing a standard technique for CNT-containing industrial wastewater treatment, and for advancing the environmental sustainability of the CNT industry.

## 1. Introduction

Due to their unique structures and outstanding electrical, thermal, optical, and mechanical properties, carbon nanotubes (CNTs), including single-walled carbon nanotubes (SWNTs) and multi-walled carbon nanotubes (MWNTs), have attracted extensive interest and demonstrated great promise in numerous application areas, such as energy storage, device modeling, sporting goods, water filters, thin-film electronics, coatings, actuators, and electromagnetic shields [1,2,3,4,5,6,7,8,9]. CNT-related industrial products are growing rapidly each year, and total worldwide production of CNTs is expected to approach 4000 tons by 2023 [10]. Most applications cannot be realized without suitable processes for dispersing CNTs in organic or inorganic solutions, preparing fibers and films, or achieving immobilization of separate particles. These processes can produce large amounts of wastewater containing CNTs. 

Additionally, there are increasing concerns due to the potential risk of CNTs to the environment and human health [11,12,13,14,15]. It was reported that CNTs can bio-accumulate in aquatic organisms [16,17] and significantly inhibit their growth [18,19,20,21]. Some studies have shown that CNTs may induce bronchoalveolar adenoma and carcinoma in mice and rats [22,23,24]. Furthermore, CNTs can persist in the body for a long time without obvious biodegradation [25,26]. In 2014, the International Agency for Research on Cancer (IARC) classified MWNT-7, a particular type of long and rigid CNT, as possibly carcinogenic to humans based on animal studies [27]. Recently, the International Chemical Secretariat (ChemSec) added CNTs to the ‘Substitute It Now’ (SIN) list of chemicals [28]. Therefore, risk management related to the safe handling, research, production, and waste treatment is important to avoid exposure to CNTs in occupational operations and the environment. Thus, CNT-containing wastewater cannot be directly discarded without treatment. 

However, there are no established methods or standard guidelines for the treatment of wastewater containing CNTs. Although filtration can remove most CNTs from aqueous systems, it can be time-consuming and it may be difficult to remove small, well-dispersed CNTs from aqueous solutions. Combustion may be useful for burning off waste from CNT-containing dispersions, but it is not feasible or affordable for dealing with large amounts of industrial wastewater from manufacturers. Recently, we discovered that CNTs can be completely degraded into carbon oxides or carbonate ions using sodium hypochlorite (NaClO) [29], which suggests that this technique could be used as a simple method for the removal of CNTs from wastewater. Hypochlorite is an inexpensive and strong oxidizing agent that has been widely used as a household disinfectant, water treatment agent, and bleaching agent since the 18th century [30]. Therefore, this method is low cost, highly effective, and safe.

We also found that most CNTs, including SWNTs, MWNTs, and carbon nanohorns (CNHs) could be completely degraded by NaClO solution. Although the degradation rates of the eleven investigated types of CNTs are different, their degradation trends are the same. The degradation rates of CNTs with NaClO mainly depend on the diameters of the different types of CNTs, ordered SWNTs ≥ CNHs > thinner MWNTs > thicker MWNTs [31], and two-dimensional graphene oxide sheets degrade faster than one-dimensional oxidized CNTs [32]. However, for practical industrial applications, more fundamental research is needed. We must clarify the effects of various factors on CNT degradation to establish an appropriate method with optimal conditions. In addition, practical wastewater contains other components besides CNTs, such as surfactants or dispersants, and these may also influence the degradation of CNTs.

In the present study, we focused on SWNTs (SG-CNTs) of high purity and medium diameter and explored the influence of various conditions and the protein bovine serum albumin (BSA), which is commonly used for dispersing CNTs [33]. The results will contribute to establishing a standard technique for the treatment of CNT-containing wastewater using NaClO.

## 2. Materials and Methods

### 2.1. Preparation of CNT Dispersions

In this study, we used SWNTs obtained using the super-growth method (SG-CNTs) [34]. SG-CNTs are single-wall carbon nanotubes with a diameter of 1–5 nm. Their density is estimated to be about 0.029 g/cm^3^ and their surface area is >800 m^2^/g [35,36]. Levels of metal impurities in samples were <0.5% [34]. To obtain CNT dispersions, SG-CNTs were dispersed in an aqueous solution of BSA (fatty acid-free; Nacalai Tesque Inc., Kyoto, Japan) following a standard process (ISO/TS 19337), as reported previously [37,38]. Briefly, SG-CNTs (50 mg) were dispersed in 50 mL of an aqueous solution of BSA (10 mg/mL) by sonication with a VC-750 homogenizer (Sonics & Materials, Inc., Newtown, CT, USA) for 3 h with cooling using an ice-water bath. The concentration of SG-CNTs in BSA solution was estimated to be ~1 mg/mL, and SG-CNT dispersions are abbreviated as SG/BSA.

To understand the influence of the dispersant, we also prepared SG-CNT aqueous solutions without any dispersant using oxidized SG-CNTs (ox-SG) as control samples. For the preparation of ox-SG, SG-CNTs (24 mg) were oxidized using 48 mL of a mixture of H_2_SO_4_/HNO_3_ (3:1) at 70 °C for 40 min. H_2_SO_4_ (98%) and HNO_3_ (70%) solutions were used as purchased (Wako 1st grade; Fujifilm Wako Pure Chem Corporation, Tokyo, Japan). After treatment, the suspensions were diluted with water and filtered through a 0.2 μm membrane to remove acids. The residue was resuspended in deionized water and filtered again. This process was repeated around five times until the pH was neutral. The obtained ox-SG samples were dispersed in deionized water using a US-1KS bath-type sonicator (SND Co., Ltd. Tokyo, Japan) for ~20 min. The concentration of ox-SG was adjusted to around 1 mg/mL.

### 2.2. Treatment of SG-CNT Dispersions with NaClO

NaClO solution was prepared by dissolving sodium hypochlorite pentahydrate (NaClO·5H2O; Tokyo Chemical Industry Co., Tokyo, Japan) in deionized water at different concentrations. To investigate the effect of NaClO concentration, 0.1 mL SG/BSA dispersions (1 mg/mL) were added to 10 mL NaClO solutions and then treated at 37 °C for 0–192 h. The concentration of NaClO was 0.22 wt%, 0.45 wt%, 1.13 wt%, 2.26 wt%, and 4.52 wt%. The concentrations of SG-CNT and BSA were around 10 μg/mL and 0.1 mg/mL, respectively.

To investigate the effect of temperature, a homogenous dispersion of 0.1 mL SG/BSA (1 mg/mL) was added to 10 mL of NaClO solution (2.26 wt%) and then treated at temperatures of 25 °C, 37 °C, 50 °C, 70 °C, and 80 °C. 

To explore the effect of pH on the degradation of CNTs, solutions of NaClO at different pH values were prepared. Acidic solutions with pH values of 3.94 and 6.88 were obtained by mixing 5 mL NaClO (4.52 wt%) with 5 mL HCl (0.45 mol/L and 0.33 mol/L, respectively). Basic solutions with pH values of 12.42 and 13.50 were obtained by mixing 5 mL NaClO (4.52 wt%) and sodium hydroxide (0.1 mol/L and 10 mol/L, respectively). A solution with pH 10.20 was prepared from NaClO (2.26 wt%) without adjustment. Next, 0.1 mL SG/BSA dispersions (1 mg/mL) were added to each solution, and mixtures were heated to 37 °C for 0–168 h (7 days).

The changes in the amounts of SG-CNTs after treatment with NaClO under different conditions was estimated by measuring the optical absorbance of CNT dispersions at a wavelength of 700 nm at each time point using a Lambda 1050 ultraviolet/visible light/near infrared (UV/vis/NIR) spectrometer (Perkin Elmer, Waltham, MA, USA) as reported previously [29,31,39]. 

### 2.3. Measurement of Free Available Chlorine in Treatments

The change in the concentration of free available chlorine (f-Cl) at each time point was determined using a portable free chlorine detector (HI96771B; HANNA Instruments, Tokyo, Japan) with a ready-made reagent (HI93701-0; HANNA instruments). This yielded the concentration of free chlorine in water samples within a 0.00 mg/L to 5.00 mg/L (ppm) range, following adaptation of USEPA Method 330.5 and Standard Method 4500-Cl G. All samples were diluted 10,000-fold for measurements. 

## 3. Results

### 3.1. The Influence of NaClO Concentrations

**Figure 1:**
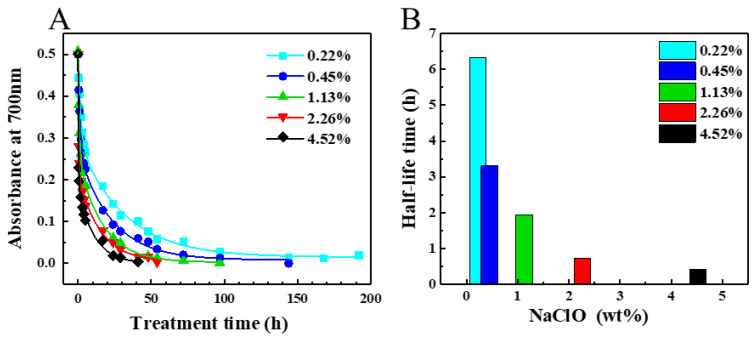
Treatment of SG/BSA dispersions at 37 °C with solutions containing different concentrations of NaClO (0.22 wt%, 0.45 wt%, 1.13 wt%, 2.26 wt%, and 4.52 wt%). (**A**) Changes in the amount of SG-CNTs during treatment, estimated from the absorbance at 700 nm. Curves were fitted using the formula Y = Y_0_ + A_1_e^−x/t1^ + A_2_e^−x/t2^ (Origin software). (**B**) The degradation half-life time of SG-CNTs. SG/BSA is a CNT-dispersion that was obtained by dispersing SG-CNTs in BSA solution (CNTs: 10 µg/mL; BSA: 0.1 mg/mL).

When the same amount of SG/BSA dispersion was mixed with different concentrations of NaClO and then treated at 37 °C, the optical absorption measurement results showed that the amount of SG-CNTs decreased with increasing treatment duration at all concentrations of NaClO (Figure 1A). The time taken for CNTs to degrade to half their original concentration, defined as the half-life time, decreased from 6.34 h to 0.43 h with increasing NaClO concentration from 0.22 wt% to 4.52 wt% (Figure 1B). However, the decrease in half-life time was not obvious when the NaClO concentration was >2.26 wt% (Figure 1B and Table 1). Complete degradation was presumed when the optical absorption of CNT dispersions at a wavelength of 700 nm was close to zero (<0.007, the absorbance of NaClO solution at 700 nm). The time taken for complete degradation of the same amount of SG-CNTs (100 μg) was also correlated with the concentration of NaClO, which was >8 days for 0.22 wt%, around 54 h for 2.26 wt%, and 41 h for 4.52 wt% NaClO, respectively (Table 1).

In addition, the amount of f-Cl in NaClO solution with/without dispersions of SG/BSA showed no obvious decrease during treatment at 37 °C after 50 h (Supplementary Information, Figure S1).

### 3.2. The Influence of Temperature

**Figure 2:**
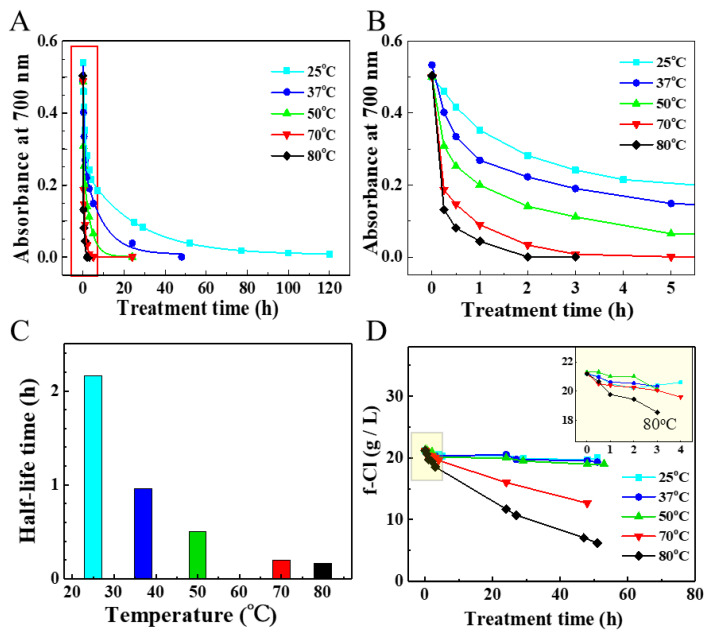
Degradation of SG/BSA with NaClO solution (2.26 wt%) at different temperatures (25 °C, 37 °C, 50 °C, 70 °C, and 80 °C). (**A**) Absorbance of SG/BSA dispersions at 700 nm during treatment, revealing changes in the amount of SG/BSA. (**B**) Close-up view of the red framed part in (**A**). (**C**) Degradation half-life of SG/BSA. (**D**) Changes in the amount of f-Cl in NaClO solution (2.26 wt%) at different treatment temperatures. The inset graph is a close-up view of the yellow framed part. SG/BSA is a CNT-dispersion that was obtained by dispersing SG-CNTs in BSA solution (CNTs: 10 µg/mL; BSA: 0.1 mg/mL).

The effect of temperature on the degradation of SG/BSA dispersions was investigated using a fixed concentration of NaClO (2.26 wt%) at various temperatures from 25 °C to 80 °C. The results indicated that degradation was temperature-dependent (Figure 2A,B). A higher treatment temperature accelerated the decrease in SG-CNTs. When the temperature was increased from 25 °C to 80 °C, the half-life of CNT degradation decreased from 2.16 h to 0.17 h (Figure 2C). The complete degradation of SG-CNTs took around five days at 25 °C, but only around 3 h and 2 h at 70 °C and 80 °C, respectively (Table 1). 

The stability of NaClO was checked by measurement of the dynamic change in the amount of f-Cl in NaClO solution (2.26 wt%) without addition of SG/BSA at different temperatures. The results showed that the amount of f-Cl did not change much when the temperature was below 50 °C, but it dramatically decreased when the temperature was increased to 70 °C or 80 °C from 0 h to 48 h. In addition, there was no decrease at 70 °C but a slight decrease at 80 °C after 3 h, at which point SG-CNTs were completely degraded (Figure 2D, inset graphs). The amounts of f-Cl remaining in NaClO solutions after 48 h were 94.3%, 92.2%, 89.2%, 59.8%, and 29.2% for 25 °C, 37 °C, 50 °C, 70 °C, and 80 °C, respectively.

### 3.3. The Influence of pH Condition

**Figure 3:**
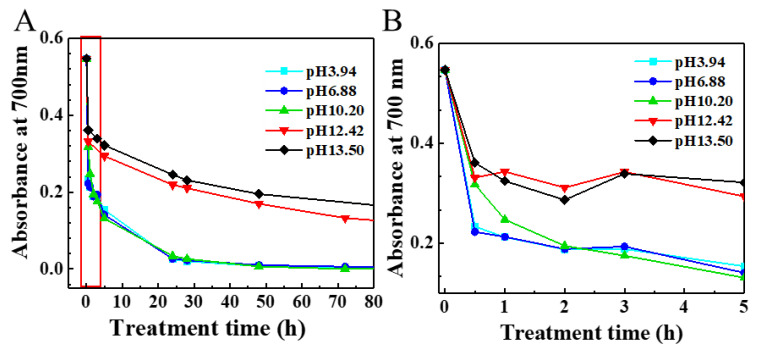
Degradation of SG/BSA by NaClO solution at different pH values. (**A**) Absorbance SG-CNTs at 700 nm in treatments with different pH values. (**B**) Close-up view of the red framed part in (**A**). SG/BSA is a CNT-dispersion that was obtained by dispersing SG-CNT in BSA solution (CNTs: 10 µg/mL; BSA: 0.1 mg/mL).

The degradation of SG-CNTs by NaClO (2.26 wt%) at different pH values was investigated using SG/BSA dispersions. Compared with NaClO solution without pH adjustment (pH 10.20), the degradation rates of SG-CNTs by NaClO solution at pH values of 3.94 and 6.88 were increased at early time points (<2 h; Figure 3A,B). The half-life of SG-CNT degradation decreased slightly from 0.82 h to 0.42 h, but the time for complete degradation did not change much (Table 1). Meanwhile, when pH values were adjusted to pH 12.42 and pH 13.50, considerably higher than that of NaClO solution (pH 10.20), the degradation of SG-CNTs slowed significantly (Figure 3A,B). The half-life time was extended to around 5 h and 15 h at pH 12.42 and pH 13.50, respectively, and SG-CNTs were not completely degraded after seven days (Table 1).

**Figure 4:**
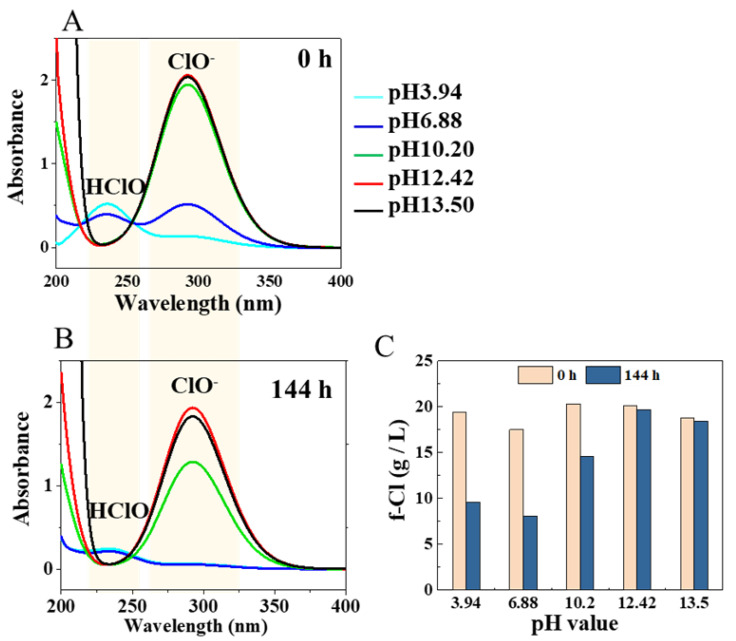
Optical absorbance spectra of NaClO solutions at different pH values at 0 h (**A**) and after 144 h (**B**) at 37 °C. (**C**) Free available chlorine (f-Cl) concentration in NaClO solutions at different pH values at 0 h and after 144 h.

The optical absorption spectra of NaClO solutions displayed a strong peak at 293 nm, indicating the existence of ClO^−^ under basic conditions (pH 10.20−13.50). Meanwhile, one peak at 230 nm under acidic conditions (pH 3.94) indicated the presence of hypochlorous acid (HClO) and two peaks at neutral conditions (pH 6.88) corresponded to ClO^−^ and HClO (Figure 4A). After treatment at 37 °C for 144 h, the peaks of ClO^−^ and HClO in the acidic solution almost disappeared, but the peak of ClO^−^ in basic solutions only decreased by around 35% at pH 10.20, and there was almost no change at pH 12.42 and pH 13.50 (Figure 4B). The f-Cl measurement results also showed that the amount of f-Cl in acidic (pH 3.94) and neutral (pH 6.88) solutions was decreased significantly, while at pH 10.20 there was a small decrease, and at pH 12.42 and pH 13.50 there was almost no change after 144 h (Figure 4C).

### 3.4. The Influence of SG-CNT Concentration and Dispersant

**Figure 5:**
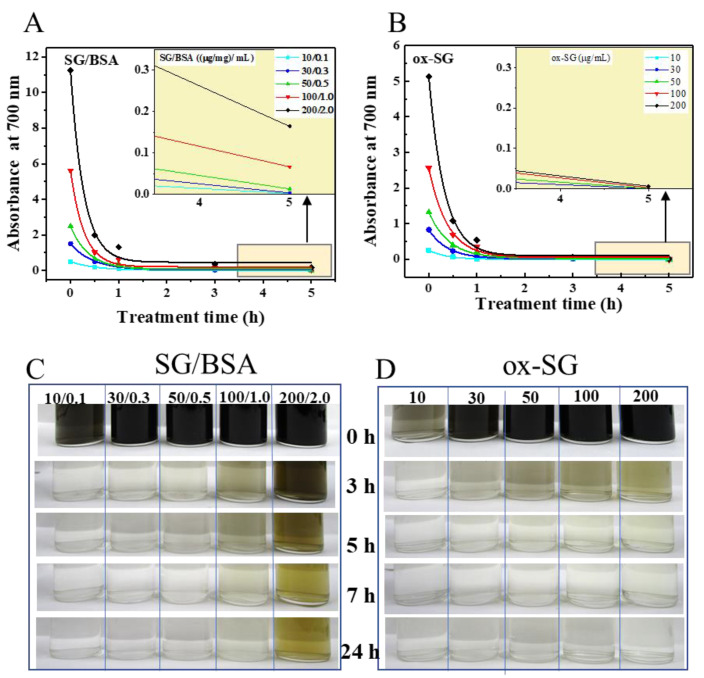
The effect of BSA dispersant on the degradation of SG-CNTs by 2.26 wt% NaClO at 70 °C. The absorbance at 700 nm indicates the change in the amount of SG-CNTs during treatment with different concentrations of SG/BSA (**A**) and ox-SG (**B**). The inset figures in A and B are close-up views of the light-yellow parts in each figure. Images of glass bottles containing SG/BSA (**C**) and ox-SG (**D**) dispersions with different concentration of CNTs following treatment with NaClO for 0−24 h. The concentrations in the legend represent the concentrations of ox-SG (µg/mL) and SG/BSA (µg or mg/mL).

To investigate the influence of other components beside CNTs, we compared the degradation of SG/BSA with BSA dispersant and ox-SG without dispersant using 2.26 wt% NaClO solution at 70 °C. The results showed that NaClO treatment decreased the amount of SG-CNTs rapidly both with and without BSA based on the optical absorbance of CNT dispersions at 700 nm (Figure 5A,B). However, we found that SG/BSA could not be completely degraded by NaClO when the SG-CNT concentration was increased to 200 μg/mL at a BSA concentration of around 2 mg/mL based on observations with the naked eye (Figure 5C) and the optical measurement (Figure 5A, inset graph), even when the observation time was extended to 24 h. By contrast, ox-SG at a CNT concentration of 200 μg/mL could be completely degraded within 7 h (Figure 5B,D), at which point the optical absorption at 700 nm was nearly zero (<0.007), and the CNT dispersion was colorless.

**Figure 6:**
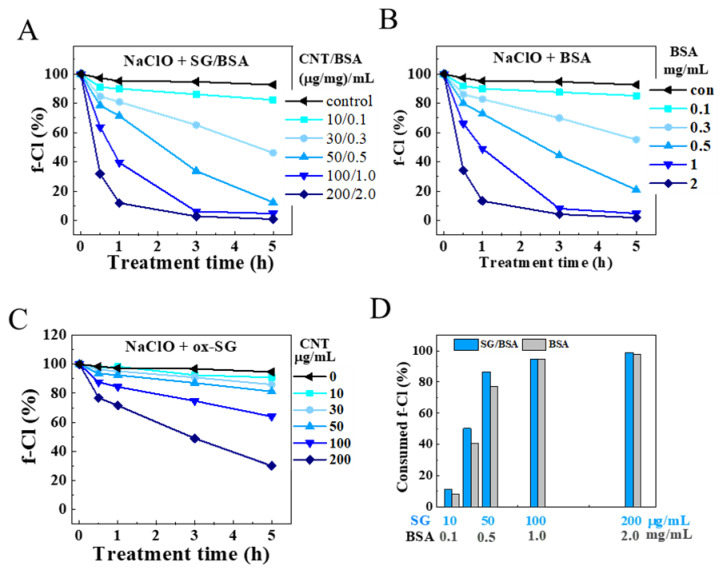
The amounts of free available chlorine (f-Cl) remaining in NaClO solutions (2.26 wt%) after 0–5 h incubation at 70 °C. (**A**) for SG/BS, in which the concentrations of CNTs/BSA were 10/0.1, 30/0.3, 50/0.5, 100/0.1. and 200/0.2 (µg/mg)/mL; (**B**) for BSA at concentrations of 0.1–2 mg/mL; and (**C**) for ox-SG at CNT-concentrations of 0–200 µg/mL. The total amounts of consumed f-Cl after five hours for SG/BSA (blue) at CNT-concentrations of 10–200 µg/mL and BSA only (grey) are shown (**D**). The concentrations of BSA in the SG-BSA samples (blue) were the same as those in the BSA samples (grey). SG/BSA dispersions were obtained by dispersing SG-CNTs in BSA solution (CNTs: 0–200 µg/mL; BSA: 0–2.0 mg/mL).

Dynamic changes in f-Cl concentration in NaClO solutions (2.26 wt%) containing SG/BSA, BSA, or ox-SG at 70 °C were measured over 5 h. The results showed that the amount of f-Cl decreased with increasing concentration of SG/BSA, BSA, or ox-SG (Figure 6A–C). The concentration of f-Cl in NaClO solution containing SG/BSA or BSA decreased significantly over time, while that of NaClO solution alone showed only a slight decrease (~7%; Figure 6A, control). The amount of f-Cl decreased by ~92% after 3 h with BSA at concentrations of 1 mg/mL and 2 mg/mL, and almost 100% of f-Cl was consumed after 5 h at a BSA concentration of 2 mg/mL (Figure 6B). When NaClO was used to treat various concentrations of SG/BSA for 5 h, the consumed amounts of f-Cl were only slightly more than those with BSA alone when it was used at the same concentrations as in SG/BSA samples (Figure 6D). This indicates that BSA was the main consumer of NaClO in SG/BSA samples under these test conditions. In addition, optical absorbance spectra showed that the absorption peak of BSA had almost disappeared after treatment for 5 h, indicating that BSA was also degraded by NaClO treatment (Supplementary Information, Figure S2). On the other hand, for ox-SG without any dispersants such as BSA, between 5% and 70% f-Cl was consumed after heating at 70 °C for 5 h at CNT concentrations of 10−200 μg/mL, much less than that in SG/BSA samples (Figure 6C).

## 4. Discussion

### 4.1. The Effect of NaClO Concentration

In theory, if CNTs are completely degraded by NaClO, via the chemical reaction C_CNT_ + 2NaClO → 2NaCl + CO_2_, then 1 mole of carbon requires 2 moles of NaClO. Therefore, the degradation of 100 µg CNTs only requires 1.24 mg of NaClO (~0.0124 wt%). This indicates that even for the lowest concentration of NaClO (0.22 wt%), NaClO was present in excess, which means that the reaction between CNTs and NaClO can be approximated as a pseudo first-order reaction. However, the results showed that the half-life times and complete degradation of SG/BSA decreased with increasing NaClO concentrations from 0.22 wt% to 4.52 wt%, indicating that the degradation of CNTs was dependent on the NaClO concentration. We also observed that when the NaClO concentration was increased from 2.26 wt% to 4.52 wt% (Figure 1B), the decrease in degradation time was not obvious. This indicates that the saturation concentration of NaClO might be ~2.26 wt%, much higher than the theoretical amount of 0.0124%. Although the degradation mechanism of CNTs by NaClO is still not fully understood, intermediate products such as oxygen-doped CNTs, graphene oxide, and aromatic compounds might be produced before complete degradation [29,31,40]; hence, the degradation of CNTs may be more complicated in practice than in theory (Supplementary Information, Figure S3). In addition, BSA in the SG/BSA dispersions may also consume NaClO, as discussed below (Section 4.4). For the rapid removal of CNTs from dispersions, an NaClO concentration of 2–3 wt% was found to be appropriate.

### 4.2. Impact of Temperature

In general, temperature influences the reaction rate: as the temperature increases, the reaction rate increases. Our results confirmed that degradation of SG-CNTs occurred at room temperature, and the degradation rate was dependent on temperature. The half-life time of CNT degradation at 80 °C was only ~0.17 h, 12.7 times shorter than that at 25 °C (2.16 h). The complete degradation of SG-CNTs was also decreased from 120 h to 2 h. This indicates that a higher temperature may be beneficial for the degradation of CNTs. However, when the temperature exceeds 70 °C, a further increase in temperature does not increase the degradation rate significantly (Figure 2C). This is due to the decomposition of NaClO at high temperatures, which results in a significant decrease in f-Cl in the NaClO solution (Figure 2D). Therefore, the appropriate treatment temperature for the removal of SG-CNTs was found to be 50–70 °C.

### 4.3. Effect of pH Values

In addition to temperature, the oxidative reactivity of hypochlorite is very sensitive to the pH of the solution. When the pH is >10, f-Cl from NaClO is in the form of ClO^−^, but when the pH is 5–10, both HClO and ClO^−^ coexist in solution, and when the pH is <5, f-Cl exists in the form of HClO and Cl_2_ [41]. This was confirmed by our measurements (Figure 4A). It is known that HClO has a redox potential (1.482 eV) almost twice that of ClO^−^ (0.81 eV), indicating a stronger oxidizing capacity [42]. However, the degradation rate of SG-CNTs under acidic or neutral conditions was not increased dramatically compared with that of NaClO solution without pH adjustment (pH 10.20; Figure 3). The main reason that acidic conditions did not significantly accelerate the oxidation rate of CNTs is presumably due to the instability of HClO, as confirmed by our optical absorption spectra and the amount of f-Cl present (Figure 4). The HClO absorption peak disappeared (Figure 4A,B), and the f-Cl concentration decreased (Figure 4C) in lower pH samples, indicating that HClO decomposed during treatment. The decomposition of NaClO under acidic conditions induced the generation of toxic chlorine gas (Supplementary Information, Figure S4), which is unfavorable for practical use. In addition, the dispersion state of SG/BSA remained almost homogenous after mixing with NaClO solution at pH 10.20, while the SG/BSA dispersion aggregated immediately when mixed with NaClO solution at pH 3.94 or pH 6.88 (Supplementary Information, Figure S5), which might also influence the degradation of CNTs [31].

In alkaline conditions, the amount of f-Cl in NaClO solution barely changes, but the oxidizing capacity of NaClO decreases with increasing pH [43]. Furthermore, when the pH of the solution was >12, the dispersion state of SG-CNTs in NaClO solution deteriorated (Supplementary Information, Figure S5), which also decreased the degradation rate of SG-CNTs. 

In summary, acidic or neutral NaClO solutions can increase the degradation rate of CNTs, but the amount of f-Cl is decreased, and toxic chlorine gas is produced from the decomposition of HClO. Alkaline conditions with pH > 12 reduced the degradation rate of NaClO significantly. Therefore, pH 10.2 is optimal for NaClO solutions, and adjustment to more acidic or basic pH is not recommended. 

### 4.4. The Influence of Dispersant of BSA

Since dispersants are often included with CNTs in CNT dispersions, we compared SG/BSA dispersions with ox-SG dispersions not containing any dispersants to investigate the effect of dispersants on the removal of CNTs from wastewater. We found that the amount of f-Cl in NaClO/BSA solutions decreased with increasing BSA concentration. When the concentration of BSA was >1 mg/mL, nearly 100% of f-Cl was consumed (Figure 6D). As a result, SG/BSA could not be degraded completely by NaClO in our test conditions when the concentration of BSA was >1 mg/mL (Figure 6). This indicates that dispersants in CNT dispersions may consume f-Cl and influence the degradation of CNTs. Therefore, it is important when treating CNT-containing wastewater with NaClO to consider the effects of other substances in the wastewater, such as dispersants. When the concentrations of these substances are high, diluting the dispersions, increasing the NaClO concentration, or supplementing with additional NaClO during treatment may be necessary.

Herein, the half-life time and time required for complete degradation of SG-CNTs were explored. It should be noted that these values may vary among different CNT types, CNT concentrations, and treatment conditions.

## 5. Conclusions

In this study, we investigated the effects of various conditions, such as NaClO concentration, treatment temperature, pH value, concentration of CNTs, and BSA dispersant, on the degradation of CNTs using NaClO solution. The results showed that temperature and NaClO concentration had the greatest impact on the degradation of CNTs. As the temperature and/or concentration of NaClO increased, the degradation rate of CNTs increased significantly. However, above a certain threshold, a further increase in temperature or NaClO concentration had little effect on the degradation rate. The optimal temperature and NaClO concentration were found to be 50–70 °C and 2–3 wt%, respectively. Lower pH can slightly accelerate the elimination of CNTs but may decrease f-Cl due to decomposition of NaClO under acidic conditions, and this may also release toxic chlorine gas. Furthermore, dispersants and other substances in the solution may consume NaClO, thereby affecting the degradation of CNTs. The results lay a foundation for establishing a standard technique for treating CNT-containing industrial wastewater using hypochlorite, which may help to prevent the release of harmful CNTs into the environment.

## Figures and Tables

**Table 1:** Time needed for SG/BSA degradation by NaClO under different conditions.

Treatment Conditions	NaClO Concentration (wt%)					Temperature					pH Values				
	(37 °C, pH: 10.20;					(NaClO: 2.26 wt%, pH10.20;					(NaClO: 2.26 wt%, 37 °C				
	SG/BSA: 10 μg/mL)					SG/BSA: 10 μg/mL)					SG/BSA: 10 μg/mL)				
	0.22	0.45	1.13	2.26	4.52	25 °C	37 °C	50 °C	70 °C	80 °C	3.94	6.88	10.2	12.42	13.5
Half-life	6.34	3.31	1.94	0.72	0.43	2.16	0.96	0.5	0.2	0.17	0.44	0.42	0.82	5	15
(h)															
Complete degradation	>8 days	~144 h	~72 h	~54 h	~41 h	~120 h	~48 h	~24 h	~3 h	~2 h	~48 h	~48 h	~48 h	>7 days	>7 days

## Data Availability

Not applicable.

## Supplementary Materials

The following are available online at https://www.mdpi.com/article/10.3390/toxics9090223/s1, Figure S1: Free available chlorine (f-Cl) concentration in NaClO solution (2.26 wt%) with or without SG/BSA (10 µg/mL) at 37 °C; Figure S2: Absorbance spectra of BSA before (red line) and after (black line) treatment with 2.26 wt% NaClO at 70 °C for 5 h; Figure S3: Scheme showing the degradation of CNTs by NaClO; Figure S4: Detection of chlorine gas from NaClO solutions after pH adjustment to acidic conditions; Figure S5: Images of SG/BSA (10 μg/mL) after treatment with 2.26 wt% NaClO at different pH values for 0–144 h at 37 °C.
