# Supplementary material for: Removal of Carbon Nanotubes from Aqueous Solutions by Sodium Hypochlorite: Effects of Treatment Conditions
Source: Toxics. 2021 Sep 16;9(9):223. doi: 10.3390/toxics9090223 (PMC8471244; doi:10.3390/toxics9090223)
Supplement: Supplementary file 1 [file toxics-09-00223-s001.zip › toxics-1364239-supplementary.pdf]

toxics

MDPI

Supplementary Materials:

Removal of Carbon Nanotubes from Aqueous Solutions by Sodium Hypochlorite: Effects of Treatment Conditions

Mei Yang, Toshiya Okazaki and Minfang Zhang*

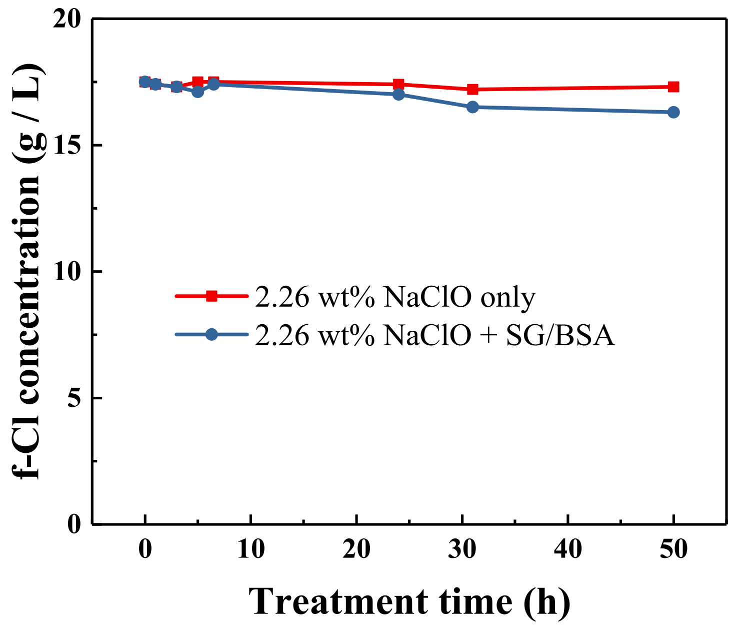

Figure S1. Free available chlorine (f-Cl) concentration in NaClO solution (2.26 wt%) with or without SG/BSA (10 µg/mL) at 37°C. **Note:** The results show that the f-Cl concentration in NaClO solution with/without dispersions of SG/BSA did not decrease obviously during treatment over 50 h.

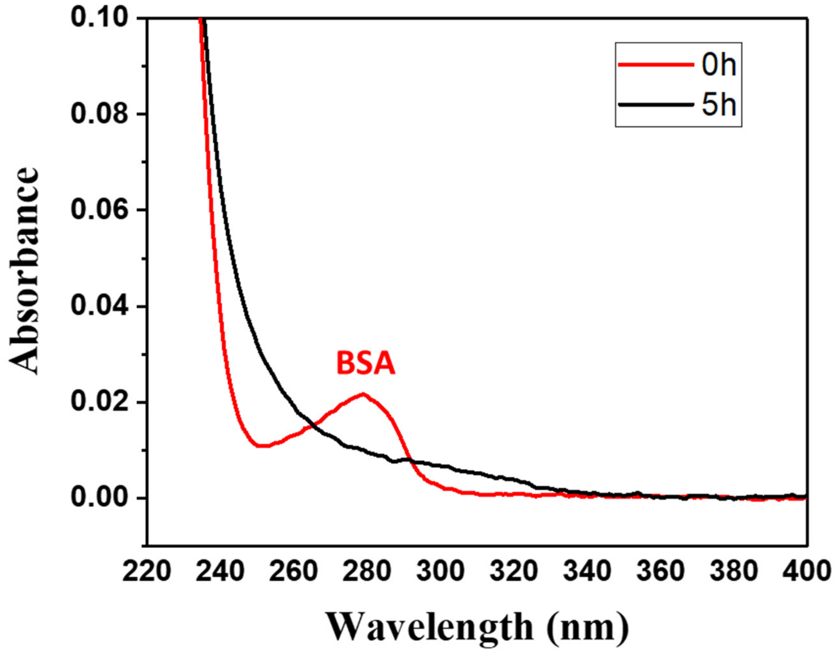

Figure S2. Absorbance spectrum of BSA before (red line) and after (black line) treatment with 2.26 wt% NaClO at 70°C for 5 h.

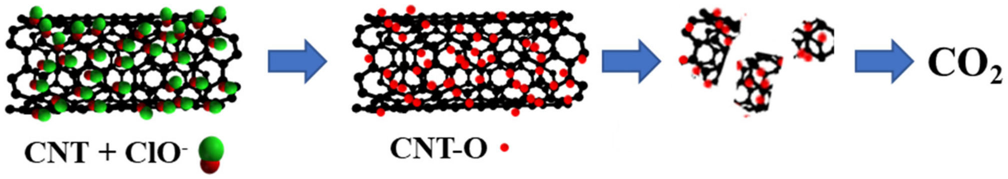

Figure S3. Scheme showing the degradation of CNTs by NaClO. **Note: From our previous study [Ref 29, 31] and other reported data [40], we propose a mechanism for the degradation of CNTs by NaClO.** When CNTs are mixed with NaClO solution, ClO^- ions donate an oxygen atom and its electrons to species being oxidized, releasing a chloride ion (Cl^-). The oxygen atoms directly attack the side walls of CNTs, forming O-doped CNTs. Continuous oxidation may lead to unzipping of CNTs, degrading them into fragments, eventually leading to complete degradation to CO_2.

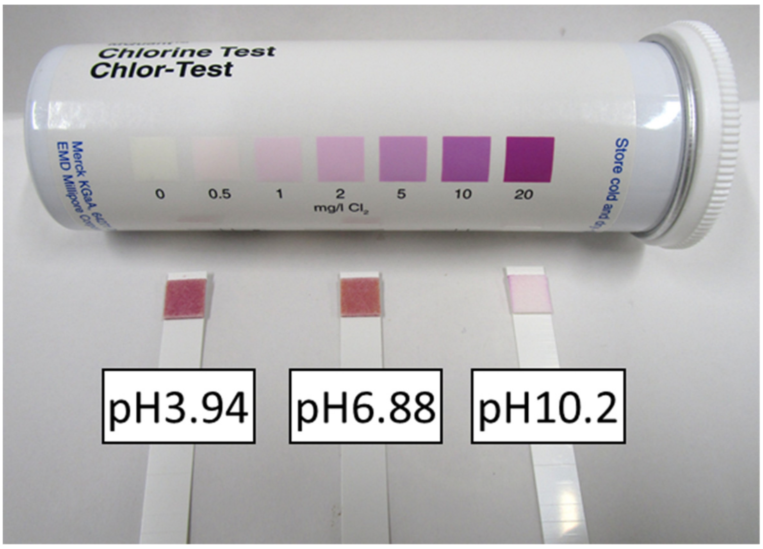

Figure S4. Detection of chlorine gas from NaClO solutions after pH adjustment to acidic conditions. Purple color on test strips indicates the generation of Cl_2. **Note: Detection was performed using a chorine test strip (Merck KGaA, Darmstadt, Germany) placed on the bottleneck of NaClO solutions for 2 s.** The test paper immediately turned purple/red for pH 3.94 and pH 6.88 groups, but only slightly purple for NaClO solution without pH adjustment (pH 10.20), confirming the generation of chlorine gas when the pH of NaClO was lowered.

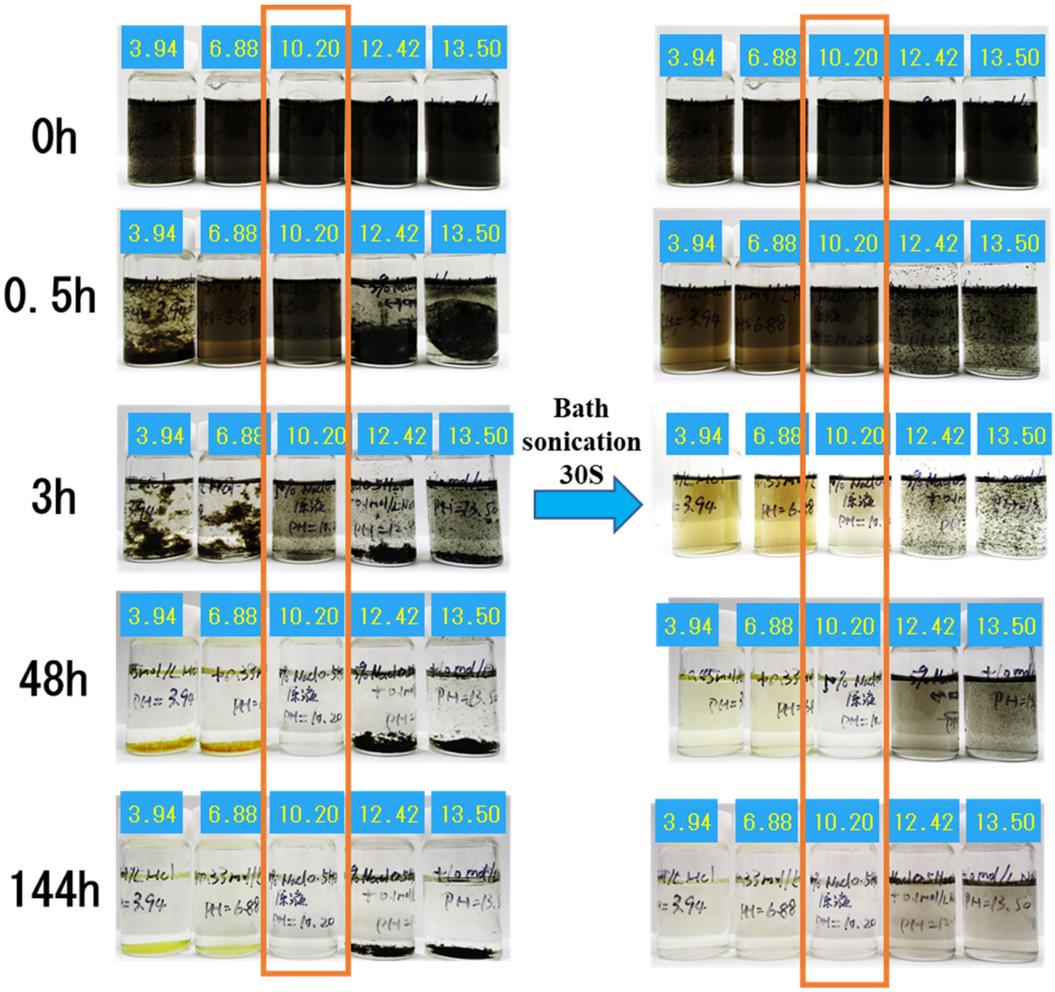

Figure S5. Images of SG/BSA (10 μg/mL) after treatment with 2.26 wt% NaClO at different pH values for 0–144 h at 37°C. SG/BSA in dispersions aggregated immediately when the pH was adjusted to more acidic (pH 3.94 and pH 6.88) or basic (pH 12.42 and pH 13.50) conditions. The dispersion state of SG/BSA in NaClO solution without pH adjustment (pH 10.20, framed in orange) barely changed.
